# Societal Narratives on Caregivers in Asia

**DOI:** 10.3390/ijerph182111241

**Published:** 2021-10-26

**Authors:** Reuben Ng, Nicole Indran

**Affiliations:** 1 Lee Kuan Yew School of Public Policy, National University of Singapore, Singapore 119260, Singapore; nicolemi@nus.edu.sg; 2 Lloyd’s Register Foundation Institute for the Public Understanding of Risk, National University of Singapore, Singapore 119260, Singapore

**Keywords:** caregivers, caregiving, narratives, social gerontology, public policy, text as data, quantitative social science, psychomics, digital humanities, aging policy

## Abstract

Although there has been an increase in awareness of the struggles experienced by caregivers, discourse on caregiving remains confined mostly to academia, policy circles or the family unit. There have been suggestions that public discourse on informal caregiving dwells overwhelmingly on the outsize toll it takes on the health of caregivers. However, few studies have examined societal narratives on caregivers—a gap our study aims to fill. We leveraged an online media database of 12 billion words collated from over 30 million articles to explore societal narratives on caregivers in six Asian countries. Computational linguistics and statistical analysis were applied to study the content of narratives on caregivers. The prevalence of societal narratives on caregivers was highest in Singapore—five times higher than Sri Lanka, which evidenced the lowest prevalence. Findings reveal that the inadequacies of institutional care as well as the need to train and empower caregivers are pressing issues that need to be prioritized on the policy agenda in Asia. Of broader significance, the diverse capabilities across Asia present opportunities for cross-country learning and capacity-building.

## 1. Introduction

The global life expectancy has more than doubled over the last hundred years—undeniably one of the greatest advancements of the 20th century. Preliminary estimates suggest that by 2030, the global population aged 80 years and above will triple between 2017 and 2050, increasing from 137 million to 425 million [1]. However, coupled with the growing burden of chronic diseases, the result is a healthcare system ill-equipped to handle the escalating demand for long-term care [2]. Amid this rapidly evolving healthcare landscape, the role of informal caregivers has assumed added prominence [3].

In many ways, caregivers constitute a vital component of the healthcare system. Physical duties aside, caregivers are tasked with responsibilities such as extending social and emotional support, liaising with various healthcare professionals, ensuring adherence to medical regimens and managing dietary needs [4]. While the value of their contributions worldwide is difficult to quantify, the approximate economic value of the services provided by caregivers in the United States totaled $470 billion in 2017 [5].

Although there has been an increase in awareness of the struggles experienced by caregivers, discourse on caregiving remains confined mostly to academia, policy circles or the family unit [6]. There have been suggestions that public discourse on informal caregiving dwells overwhelmingly on the outsize toll it takes on the health of caregivers [7]. However, there are few studies on how issues related to caregivers are discursively constructed in the media—a gap our study aims to close.

The narratives surrounding caregivers merit attention in the following aspects. First, caregivers will reconstruct their identities based on their perception of how society views and discusses issues related to caregiving [6]. These narratives have the potential to either legitimize or invalidate their identities, which may in turn affect their ability to provide sustained care. Second, narratives on caregivers are bound to impact the other half of the caregiving dyad: the care recipient. Prevailing societal conceptions of caregiving will have profound ramifications on how care recipients make sense of their need for care, the caregiving relationship and ultimately their sense of self. Third, most individuals will become caregivers or care recipients. Narratives on caregivers will determine one’s attitudes towards caregiving and consequently, how well one transitions into the role of a caregiver or care recipient.

We focus on the societal narratives surrounding caregivers in Asia for several reasons. First, while many countries are grappling with the rising demand for long-term care, the issue is particularly acute for Asian countries, where the responsibility of caregiving generally falls within the purview of the family [8]. Hence, in contrast to European states, which have long implemented long-term care policies, provisions for care are fairly new on the policy agenda in many Asian societies [8]. Second, Asia is among the most rapidly aging parts of the world [9]. In a context of reduced family sizes and increased participation of women in the workforce, the supply of caregivers—most of whom are women—is fast diminishing, thus jeopardizing the ability of the family to be the central source of care. Third, the benefits and stresses of caregiving—albeit universal—are exceptionally unique for Asian caregivers, for whom the task of providing care is a cultural norm. Past studies on how norms of filial obligation inform caregiving outcomes have yielded mixed findings. While some contend that filial obligation is associated with improved mental and physical well-being of caregivers, others find the contrary holds true [10]. Caregivers may also find themselves trapped in a ‘caring dilemma’, where they are motivated by culture to sustain their caregiving practices but simultaneously resent the unavoidability of their plight [11,12].

Our study is conceptually and practically significant. While there has been a fair amount of research on the narratives of caregivers themselves, much less is known about societal narratives on caregivers. This study serves as a marker of where caregivers stand in Asian societies. The various narratives on caregivers will shed light on how countries in Asia attempt to maintain their intergenerational cycle of care against a backdrop of sociodemographic changes threatening to erode the primacy of the family in providing care. The comparative nature of our approach also allows us to identify how countries fare relative to each other as well as on a regional basis, thus laying the groundwork for cross-country learning. Second, through exploring the narratives pertaining to caregivers, we identify the themes that dominate public discourse on caregivers as well as those given less attention. From a practical lens, this study provides crucial insights into what could be prioritized on the policy agenda, functioning as an evaluative toolkit that will guide policymakers in managing issues regarding caregivers.

## 2. Materials and Methods

### 2.1. Dataset

With 12 billion words, the News on the Web corpus is the largest known international corpus to date. It comprises over 30 million newspapers and magazines collected from 7000 websites across 20 countries [13]. This dataset was funded by the National Science Foundation and the National Endowment for the Humanities as part of studying contemporary language usage in countries where English is commonly used, making it well-suited to our study. Cultivation theory [14] indicates that the huge representation of online media accurately captures societal narratives of various countries, thus making the corpus a powerful tool for analysis. For this study, we used a full year’s (2017) dataset of 1.75 billion words. Data in the corpus include six countries/cities: Sri Lanka, India, Malaysia, Singapore, Hong Kong and the Philippines.

Each country/city is represented by an extensive variety of news sources. News sources from Sri Lanka include Ada Derana, Sunday Observer and Sri Lanka Guardian, while those from India include The Pioneer, The Statesman and Times Now. The Standard and Hong Kong Free Press are among the sources which make up the Hong Kong corpus. Sources from the Philippines include The Philippine Star, Philippine News Agency and SunStar. News outlets such as The Star, The Sun Daily and Malay Mail represent Malaysia. Singapore is represented by sources such as The Straits Times, Channel News Asia and Today.

### 2.2. Measurement of Narratives on Caregivers and Analytic Strategy

For the target words ‘caregiver’ and ‘caregivers’ in each country, we compiled the top 200 words (collocates) that co-occurred most frequently. These target words were selected for two reasons. First, ‘caregiver(s)’ had the highest prevalence globally. Second, ‘caregiver(s)’ was used most commonly across the six countries. The prevalence of narratives on caregivers in the dataset was calculated by the ratio of the number of times the target words appeared in the respective country’s dataset (numerator) and the total number of words available in the respective country’s dataset (denominator). The ratio was multiplied by 1,000,000 to provide the prevalence rate per million. 

The collocates were compiled based on the following criteria: (a) Lexical Proximity: collocate present within four words before or after the respective target word. Articles such as ‘the’ and ‘a’ were not included in the six-word lexical span. If the target noun (e.g., caregiver) was the first word of a sentence, the collocates from the preceding sentence were excluded; (b) Relevant Context: collocate referred specifically to caregiver(s) (checked by two raters); (c) Mutual Information Score of three and above: collocate had a stronger association with the target word than other words in the corpus for that country, indicating semantic bonding [15]. This is an application of concordance analysis, utilized in computational linguistics to explore the evolution of narratives [16,17,18,19,20,21,22,23,24]. Latent Dirichlet Allocation (LDA)—a technique for topic modelling—was performed on the collocates. Thereafter, we generated a list of topics for each country to identify societal narratives on caregivers.

## 3. Results

### 3.1. Size of Conversations

**Figure 1:**
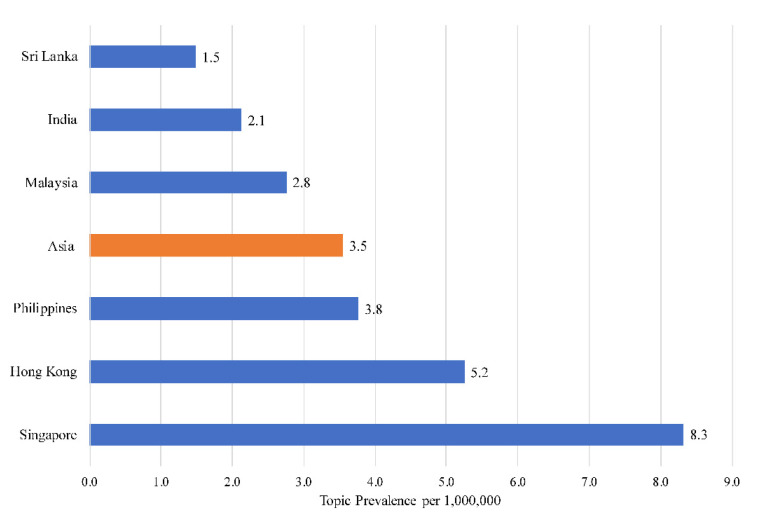
Prevalence of societal narratives on caregivers across six Asian countries/cities.

Asia averaged a baseline of 3.55 words per million. Singapore had the most narratives on caregivers (8.32 words per million), while Sri Lanka had the fewest (1.48 words per million). The sizes of conversations can be found in Figure 1, and the topics that make up the societal narratives on caregivers are in Table 1.

### 3.2. Societal Narratives on Caregivers across Six Asian Countries

#### 3.2.1. Asia

Forty percent (40%) of the narratives in Asia were on caregiver training and empowerment. Another 40% were on specialized and institutional care. In total, 10% of the narratives pertained to either foreign or family caregivers, and the remaining 10% contained miscellaneous topics. Supplementary Figures S1–S6 present a visualization of words that frequently co-occurred with ‘caregiver(s)’ in the relevant country.

#### 3.2.2. Sri Lanka

**Figure 2:**
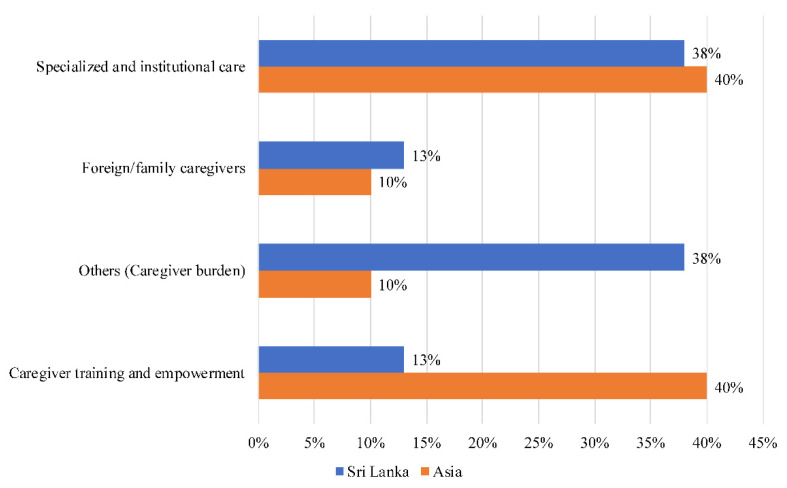
Societal narratives on caregivers in Sri Lanka.

A key narrative in Sri Lanka (Figure 2) pertained to caregiver burden (illness, depression, suffer). This accounted for 38% of the total narratives, which was above the Asian average of 10%. Another 38% of narratives were on specialized and institutional care (organization, provision, physician). Only 11% of the narratives were on caregiver training and empowerment (development, facilitate, awareness), compared to the average of 40% in Asia. Topics concerning foreign caregivers (Italian, northern, memorandum) accounted for 11% of the total narratives.

#### 3.2.3. India

**Figure 3:**
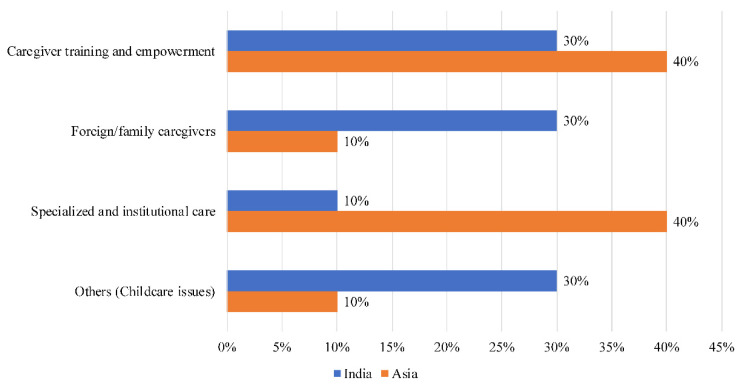
Societal narratives on caregivers in India.

Thirty percent (30%) of the narratives in India (Figure 3) were on family caregivers (family, role, support), 30% were on caregiver training and empowerment (training, support, program) and another 30% were on issues related to childcare (infant, child, scheme). Childcare-related topics were present only in India. The last 10% of narratives were on specialized and institutional care (hospital, patient, visit), which was far below the Asian average of 40%.

#### 3.2.4. Hong Kong

**Figure 4:**
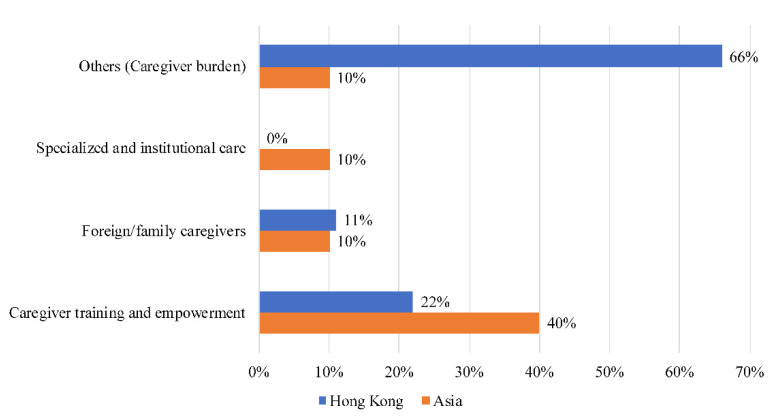
Societal narratives on caregivers in Hong Kong.

Sixty-six percent (66%) of narratives were about caregiver burden (stress, toll, mental) in Hong Kong (Figure 4)—the highest of the six countries and more than six times the Asian average. In total, 22% of narratives were related to caregiver training and empowerment (support, organization, volunteer). Topics concerning foreign caregivers (Filipino, foreign, worker) accounted for 11% of the total. In contrast to the rest of Asia, there were no topics concerning specialized and institutional care in Hong Kong.

#### 3.2.5. Philippines

**Figure 5:**
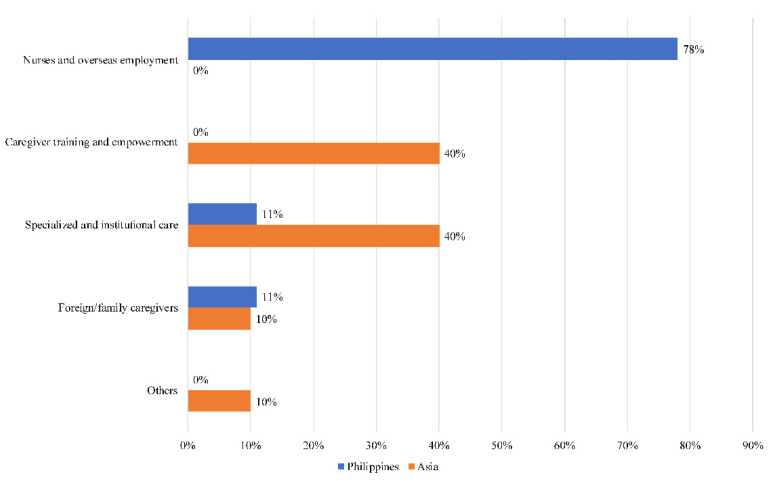
Societal narratives on caregivers in the Philippines.

Only in the Philippines (Figure 5) did the topic of nursing training and overseas employment surface. Seventy-eight percent (78%) of narratives centred around this topic (Filipino, abroad, work). In total, 11% of narratives were regarding specialized as well as institutional care (hospital, professional, worker), while the remaining 11% were on family caregivers (parent, partner, spouse). Unlike in the rest of Asia, there was no discussion on caregiver training and empowerment in the Philippines.

#### 3.2.6. Malaysia

**Figure 6:**
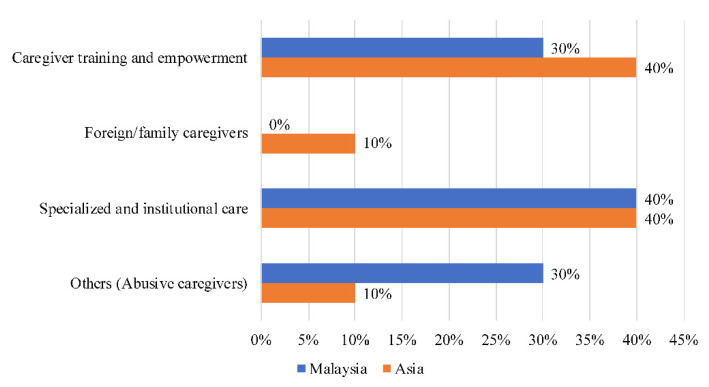
Societal narratives on caregivers in Malaysia.

Forty percent (40%) of narratives on caregivers in Malaysia (Figure 6) were related to specialized and institutional care (hire, employ, recruit). In total, 30% of narratives revolved around abuse by caregivers (abuse, report, parents)—a theme which appeared only in Malaysia. Another 30% of narratives focused on caregiver training and empowerment (support, training, education).

#### 3.2.7. Singapore

**Figure 7:**
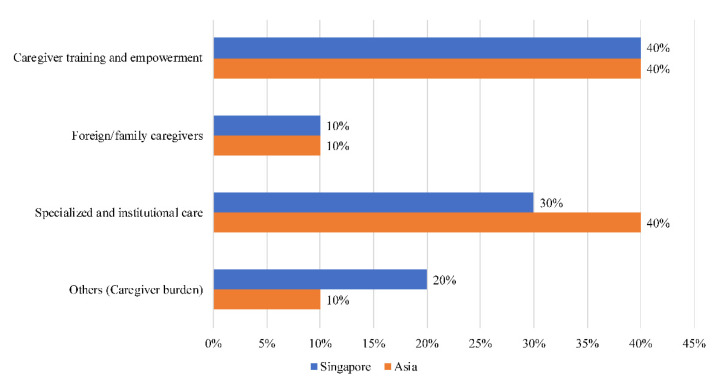
Societal narratives on caregivers in Singapore.

Forty percent (40%) of narratives on caregivers in Singapore (Figure 7) pertained to caregiver training and empowerment (support, training, initiative)—the highest of the six countries. In total, 30% of narratives were regarding specialized and institutional care (voluntary, welfare, center). Another 20% of narratives were on caregiver burden (burden, stress, challenge), and 10% were on family caregivers (parent, compassionate, caring).

## 4. Discussion

This is one of the first known studies on societal narratives on caregivers in Asia. These narratives reveal the key issues that are foregrounded and underdiscussed in society, providing insights into what could be prioritized on the policy agenda moving forward. We discuss the conceptual significance of our findings across Asia.

### 4.1. Sri Lanka

Relative to countries at a similar level of economic development, Sri Lanka’s transition to an aging society is taking place at an accelerated pace, occurring at a speed not unlike countries with a much higher per capita income [25]. The share of the population aged 65 and above will shift from 9.4 percent in 2015 to 21 percent in 2045 and eventually to 35.6 percent in 2100 [25]. Despite this, narratives on caregivers in Sri Lanka are dismally few and the number significantly below the regional average—the lowest of the six countries. In the rare instances that caregivers are discussed, narratives centre mostly on institutional care as well as caregiver burden. Although the family has historically been the mainstay of care [26], higher levels of migration among younger family members have led more to send their aging parents to long-term care facilities [27]. 

Similar to other developing countries with scarce resources, institutional care in Sri Lanka is severely limited [26]. The long-term care facilities in Sri Lanka that do exist suffer from a severe shortage of qualified and trained healthcare staff. This has led to a prevalence of errors in the prescription, administration and storage of medication—errors which untrained caregivers are unable to detect [27]. It is therefore a major cause for concern that the topic of training caregivers accounts for only a tiny fraction of the narratives. The prevalence of topics pertaining to caregiver burden further underscores the need for society to have more discussion on caregiver training and empowerment to enable caregivers to become more adept at both providing care and managing caregiver burden.

### 4.2. India

India has the second-lowest prevalence of narratives on caregivers. There is little discussion on specialized and institutional care in India—an expected finding, given the culturally prescribed nature of the caregiving role [28] as well as the dearth of long-term care infrastructure in the country [29]. As in many other countries, there has been an appreciable drop in the number of available caregivers in India due to rising life expectancies and decreasing fertility rates. Moreover, the increase in migration of young people overseas for better educational and professional prospects [29] is slowly resulting in the demise of the extended family system, undermining support for older adults. The country therefore appears underprepared to meet the challenges of its aging population. However, rather than expand institutional support, the government has resorted to legislating the care of older adults by children, heirs or relatives [30].

Fortunately, there is evidence that both intranational and transnational caregiving are beginning to gain currency in India [29]. While international migration may threaten to sever the obligation to provide care, care work continues to be performed across borders through the affordances of technology. This is a heartening trend, although institutional support ought to be strengthened to prevent caregiver burnout in the long run. 

Only in India did the topic of childcare emerge. This could be a result of the strong emphasis placed by the government on childcare in national policies and schemes [31].

### 4.3. Hong Kong

Hong Kong boasts the longest life expectancy of any territory and country in the world [32]. However, its fertility rate remains the lowest of the six countries, with its total number of births per women plummeting to a historic low of 0.87 in 2020 [33]. Although expectations of caregiving in Hong Kong are shaped by mores such as filial piety, over the years the rise in the set-up of the nuclear family has weakened the support extended to older adults. Hence, older people in Hong Kong are institutionalized at a high rate relative to those in Singapore and Japan [34]. The government has been criticized for failing to assist caregivers in overcoming structural obstacles such as growing monthly expenses and long working hours [35]. In fact, a recent study found that a quarter of caregivers who had to look after their aging family members had three psychosocial health problems at the same time [36]. It is thus unsurprising that narratives on caregivers in Hong Kong are monopolized by the topic of caregiver burden. Hong Kong has also been said to be lagging behind other countries in terms of provision of support for caregivers [37], which is reiterated by the lack of narratives on caregiver training and empowerment. 

There is a pressing need to improve the quality of institutional care—a topic which generated absolutely no discussion. In Hong Kong, institutionalization is deemed a last resort not only because of the desire to age in place, but also due to concerns regarding poor quality of care, the attitude of staff and a perceived loss of dignity [38]. Furthermore, the inability of long-term care facilities to cope with the surge in the number of older adults has affected the quality of service provided, with waiting times for government-subsidized residential homes taking up to three years [38]. Even as the home remains the preferred locus of care, it is critical that more conversations regarding institutional and community-based care are generated so that improving care systems becomes prioritized in the policy agenda. This will ensure that older persons are able to age with dignity and caregivers are not overburdened.

### 4.4. Philippines

As expected, discourse on caregivers in the Philippines revolves predominantly around nurses and overseas employment. The country is one of the biggest international suppliers of nurses and domestic labour [39]. Concerns over a growing crisis of care have long been flagged, with the country losing its trained and skilled nursing workforce much faster than the workers can be replaced [40]. Additionally, there are worries over a care deficit for children whose mothers work overseas as nurses or domestic helpers [34]. Narratives on caregiver training and empowerment are curiously absent in the Philippines. Research has documented the difficulty of mothering from a distance [41], which accentuates the need to provide caregivers based overseas with greater support.

Discussion on institutional care in the country is also noticeably limited. Filipino caregivers depend mainly on family resources when providing care and deliberately refrain from using formal or community-based services. As a matter of fact, those who rely on these services are deemed unable to provide adequate care and therefore stigmatized [42]. Hence, the few caregivers who utilize formal support do so in secret to avoid being socially discredited [42]. Even as family-centred caregiving remains the key mode of care, culturally sensitive services should be developed to mitigate caregiver strain [43].

Although the Philippines is not aging as rapidly as other parts of Asia—it is the youngest of the six countries [44]—the country is nonetheless confronting other sweeping sociodemographic phenomena such as an increase in labour force participation among females, increasing life expectancies and declining fertility rates [45]. It would therefore be prudent for policymakers in this country to employ a systematic, pre-emptive approach in tackling the eventual increase in long-term care needs.

### 4.5. Malaysia

The number of narratives on caregivers in Malaysia is below the Asian average. It is also the only country where the theme of abusive caregivers emerged. Prior research has established that the abuse of older people by caregivers is on the rise in Malaysia—an alarming trend that has resulted in calls to improve legislation to protect older adults from abuse [46]. Caregiver burden is often a prelude to abusive behaviour [47], which signals the importance of programs to support and equip caregivers with skills to manage the onerous task of providing care. 

Like Sri Lanka, there have been concerns about the quality of care administered by caregivers in Malaysia. Care centres and nursing homes have been said to lack discernment in hiring caregivers, with many hiring untrained staff [48]. In recent years, however, organizations such as CARE Concierge and Aged Care Group have been set up to provide caregivers with the skills needed to deliver proper care [49].

Due to limitations in formal care provision as well as norms concerning filial obligations, the family generally retains a salient role in looking after older family members [50]. However, the high levels of caregiver burden have sparked greater discourse on the need to offer more specialized and institutional care. Despite the fact that the Malaysian government offers institutional and home-care support for long-term care recipients, a recent study found that informal care providers mainly receive support from family members, with little support from professional, day or institutional care systems due to the limited availability of these services [51].

### 4.6. Singapore

Singapore is a noteworthy outlier in this study; its GDP per capita far exceeds that of the remaining five countries [52]. Narratives on caregivers are the most prevalent in Singapore. Compared to the other countries, discussions related to caregiver training and empowerment are prevalent in Singapore, presumably owing to the country’s relatively comprehensive long-term care system [53]. Various initiatives—in the form of grants and training courses—have been put in place to support caregivers in their care trajectory [54], which could account for the relatively few narratives centred around caregiver burden.

Discourse on specialized and institutional care is fairly substantial, likely due to dwindling fertility rates as well as increasing life expectancies—both of which have propelled the government to boost the capacity of the intermediate and long-term care sector [54]. Guided by a framework that seeks to allow older adults to ‘age in place’, the Singapore government has piloted various models of care in order to create a proactive and broad-based community care system [55].

Worth noting is that despite the growing range of institutional or community-based facilities and services in Singapore, the costs have been prohibitive for some [54]. This has led to calls for greater public spending in community-based and home-based care to relieve the burden of cost on both care recipients and providers [56].

### 4.7. Pan-Asian Societal Narratives on Caregivers

Even as narratives on caregivers in the various Asian societies converge in several ways, they must be understood in a context broad enough to cover the social, economic and demographic conditions unique to each country. Practitioners from each of the six countries can look to other countries—as benchmarks, role models or even cautionary tales—to engage in collaborative and cross-country learning.

Currently, there is limited discourse on specialized and institutional care in both India and the Philippines, since family-centred care remains the dominant mode of care provision in both countries. Although both countries are not aging as rapidly as the others, both lag behind in terms of GDP growth [52]. As fertility rates inch downwards and life expectancies rise, it will only be a matter of time before the financial pressures imposed by an aging population become palpable. More discourse must be generated on how to manage the imminent increase in long-term care needs under the constraints of limited economic resources so that the demands of long-term care do not outpace the speed of economic growth. Countries such as Singapore and Japan make useful points of reference, with both having pivoted towards developing community-based models of care to meet the rising demands of long-term care in an economically viable manner [55,57]. 

To better support individuals in fulfilling their caregiving responsibilities, more resources need to be channelled into creating long-term facilities with well-trained staff in both India and the Philippines. In this respect, the substandard quality of long-term care facilities in Sri Lanka can be looked to as a cautionary tale. Ensuring that caregivers are adequately trained will prevent older adults’ existing health conditions from being exacerbated by errors in prescribing or dispensing medication as have occurred in Sri Lanka [27].

Discussions on specialized and institutionalized care did not surface in Hong Kong despite the glaring structural deficiencies in the country’s long-term care system. Although Hong Kong has attempted to implement community-based models of care like those in Singapore and Japan, community care services remain insufficient. Singapore and Japan serve as more realistic benchmarks for Hong Kong and Malaysia, as all four are relatively advanced economies that are aging rapidly. Reputed for having an efficient healthcare system, Singapore may display a standard that Hong Kong can seek to emulate. More targeted initiatives can be implemented at the community level to ensure that institutional facilities are not overwhelmed by the demands of long-term care.

Discussions of caregiver burden account for the bulk of public discourse in Hong Kong and Sri Lanka—a reflection of the reality of caregiver burnout in these countries. It is crucial that the challenges of caregiving are acknowledged and discussed. Such narratives aid in capturing the often stressful nature of caregiving and offer caregivers much-needed validation. However, these narratives may produce unintended consequences when they take centre stage. Existing caregivers might harbour only the negative aspects of caregiving, which could have deleterious effects on their psychological well-being. Likewise, potential caregivers could be discouraged from accepting the caregiving role if they anticipate it as an arduous and distressing undertaking. Care recipients might also construe their own need for care as burdensome and oppressive, which could lead to feelings of guilt and a reduced sense of self [58].

In contrast, the topic of caregiver burden did not surface in the narratives in India and the Philippines. This should not be taken to mean that caregivers in these countries do not face caregiver burnout. Instead, we argue that this attests to the enormous dedication to caregiving among carers in India and the Philippines. Rather than diminishing the capabilities of families to support their aging parents, structural changes have given rise to new and innovative forms of caregiving in India, as epitomized by the country’s ‘caregiving from a distance’ model [29]. In the same vein, individuals in the Philippines continue to provide care even after migrating to and assimilating into other societies [43]. Moreover, although gender did not surface as a key theme in the narratives, caregiving is a role predominantly undertaken by females in all the countries explored in this study except the Philippines. In the Philippines, both males and females partake in the caregiving process. For instance, it is not uncommon for males to assist with tasks that are more physically demanding, such as household chores [43]. With female labour force participation increasing in societies worldwide, it is crucial that the duty of providing care does not fall squarely on women’s shoulders. The remaining countries can therefore look at both India and the Philippines as model exemplars with regard to their commitment to caregiving.

Uplifting accounts of the caregiving experience are conspicuously absent in all six countries. To encourage individuals to take on the caregiving role, positive experiences of the caregiving journey could be highlighted as a powerful counterbalance to the negative side of caregiving that typically pervades public discourse. This will eradicate the notion that providing care is a taxing, overwhelming and even dangerous task [7] and advance the idea that caregivers are compassionate, resilient and family-oriented individuals without whom the healthcare system would collapse.

Many individuals enter the caregiving role without any formal preparation and with limited knowledge, resources and skills [59]. It is therefore a welcome step forward that Singapore is having more discussions about training and empowering caregivers. Caregiver training has been said to bolster self-efficacy, relieve anxiety in providing care and prevent caregiver burnout—all while improving the quality of life of care recipients [60]. However, discussions on these topics in the remaining countries are exceedingly few. Recently, countries such as Hong Kong and Sri Lanka have made inroads in the training of caregivers [61,62]. Even as these represent great strides, efforts to address the needs of caregivers should take on a more holistic and less piecemeal approach.

Although limited, narratives on foreign caregivers are present in a few of these countries. Outsourcing care work to foreign domestic helpers has become a trend in many societies. However, despite their immense contributions, by and large, foreign care workers inhabit a marginal social position in host societies [41]. Poignantly, some Filipino medical workers feel shame about themselves and the nation due to its association with low-status feminized labour [63]. The role of foreign domestic workers is certain to grow in importance in the foreseeable future. This will come at the expense of an increase in caregiver burden. Under such circumstances, policy coordination across national borders is essential [53] and will create opportunities for cross-country learning and capacity-building.

The limitations of this study provide ideas for future research. Our database did not include social media—a potential source of narratives—although the diversity of social media usage across multiple platforms would render data collation challenging. Moreover, most social media platforms such as Facebook are closed for public access. They have also become increasingly monetized, selling datasets that may not be representative. Nevertheless, this is a significant drawback that we will overcome when the database is augmented in future studies that could complement existing methods [64,65,66,67,68,69,70,71,72] and triangulate findings across cultures [73,74,75,76,77,78,79,80,81] using multiple sources. 

## 5. Conclusions

In Asia, caring for an older loved one is so culturally ingrained that the struggles of caregivers tend to go either unnoticed or unaddressed by society. Meeting the ever-growing demands for long-term care necessitates a multi-sectorial approach which sees individuals, the community and the state working in concert. We hope that policymakers and non-government organizations will take heed of the pressing need to address the inadequacies of institutional care and the lack of support for caregivers. These issues should be at the top of the policy agenda in Asia. On a positive note, the diverse strengths across long-term care systems in Asia present opportunities for cross-country learning and capacity-building.

## Figures and Tables

**Table 1:** Topics and associated keywords of societal narratives on caregivers across six Asian countries/cities.

Country	Topic	Collocates (Keywords)
Sri Lanka	Specialized and institutional care	*Organization, provision, physician, diagnosis, knowledge, information, sufficient*
	Caregiver burden	*Illness, depression, suffer, health, condition, disabling, work, huge, sole*
	Foreign caregivers	*Italian, northern, memorandum, profession, nursing, sector, midwifery, dearth, severe*
	Caregiver training and empowerment	*Development, facilitate, awareness, raise, better, sole, demand, suffer, target*
India	Caregiver training and empowerment	*Training, support, program, learn, share group, discussion, experience, education, information*
	Family caregivers	*Family, role, support, care, parent, rule, govern, social, Indian, loved*
	Childcare issues	*Infant, child, scheme, patient, abuse, report, help*
	Specialized and institutional care	*Hospital, patient, visit, staff, nurse, stay, require*
Hong Kong	Caregiver burden	*Stress, toll, mental, health, relationship, family, psychiatrist, complain, alarming, lack*
	Caregiver training and empowerment	*Support, organization, volunteer, care, recruit, family, selfless, stress, parent*
	Foreign caregivers	*Filipino, foreign, worker, care, center, organizer, resource*
Philippines	Nurses training and overseas employment	*Filipino, abroad, work, nurse, domestic, hire, care, child, job, worker*
	Specialized and institutional care	*Hospital, professional, worker, patient, care, robot, support*
	Family caregivers	*Parent, partner, spouse, relative, mother, designated, learn*
Malaysia	Caregiver training and empowerment	*Support, training, education, course, public, attend, learn, health, care, train*
	Specialized and institutional care	*Hire, employ, recruit, private, nurse, facilitate, care, qualify, technology, implementation*
	Abusive caregivers	*Abuse, report, parent, incident, difficult, trained, increase, more*
Singapore	Caregiver training and empowerment	*Support, training, initiative, share, experience, information, volunteer, train*
	Specialized and institutional care	*Voluntary, welfare, center, association, resident, support, professional, patient, organize, cost*
	Family caregivers	*Parent, compassionate, caring, care, loved, special, elderly*
	Caregiver burden	*Burden, stress, challenge, condition, mental, illness, pressure, encourage, support*

## Data Availability

Data are publicly available at https://www.english-corpora.org (accessed since 1 January 2015).

## Supplementary Materials

The following are available online at https://www.mdpi.com/article/10.3390/ijerph182111241/s1 Figure S1. Word cloud which visualizes the words that co-occurred most frequently with ‘caregiver(s)’ in Sri Lanka; Figure S2. Word cloud which visualizes the words that co-occurred most frequently with ‘caregiver(s)’ in India; Figure S3. Word cloud which visualizes the words that co-occurred most frequently with ‘caregiver(s)’ in Hong Kong.; Figure S4. Word cloud which visualizes the words that co-occurred most frequently with ‘caregiver(s)’ in the Philippines. Figure S5. Word cloud which visualizes the words that co-occurred most frequently with ‘caregiver(s)’ in Malaysia. Figure S6. Word cloud which visualizes the words that co-occurred most frequently with ‘caregiver(s)’ in Singapore.
