# Supplementary material for: Societal Narratives on Caregivers in Asia
Source: Int J Environ Res Public Health. 2021 Oct 26;18(21):11241. doi: 10.3390/ijerph182111241 (PMC8583461; doi:10.3390/ijerph182111241)
Supplement: Supplementary file 1 [file ijerph-18-11241-s001.zip › ijerph-1401589-supplementary.pdf]

Supplementary Material

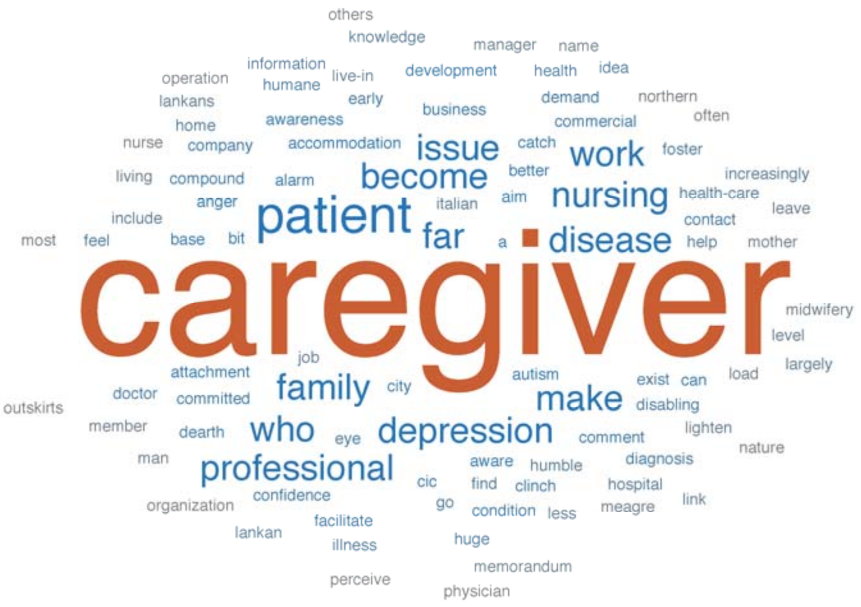

Figure S1. Word cloud which visualizes the words that co-occurred most frequently with 'caregiver(s)' in Sri Lanka.

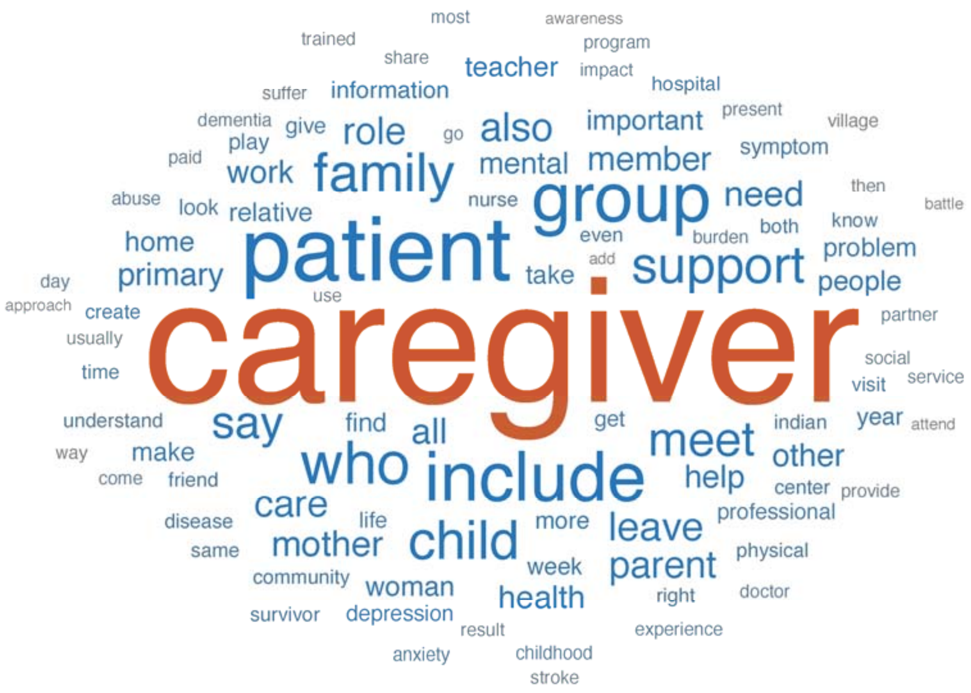

Figure S2. Word cloud which visualizes the words that co-occurred most frequently with 'caregiver(s)' in India.

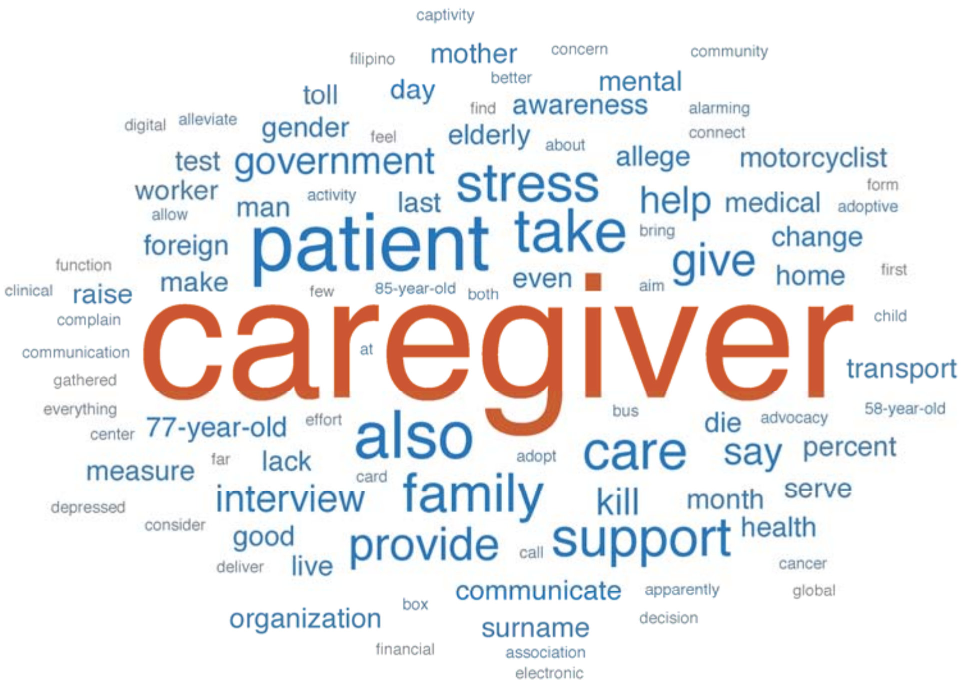

Figure S3. Word cloud which visualizes the words that co-occurred most frequently with 'caregiver(s)' in Hong Kong.

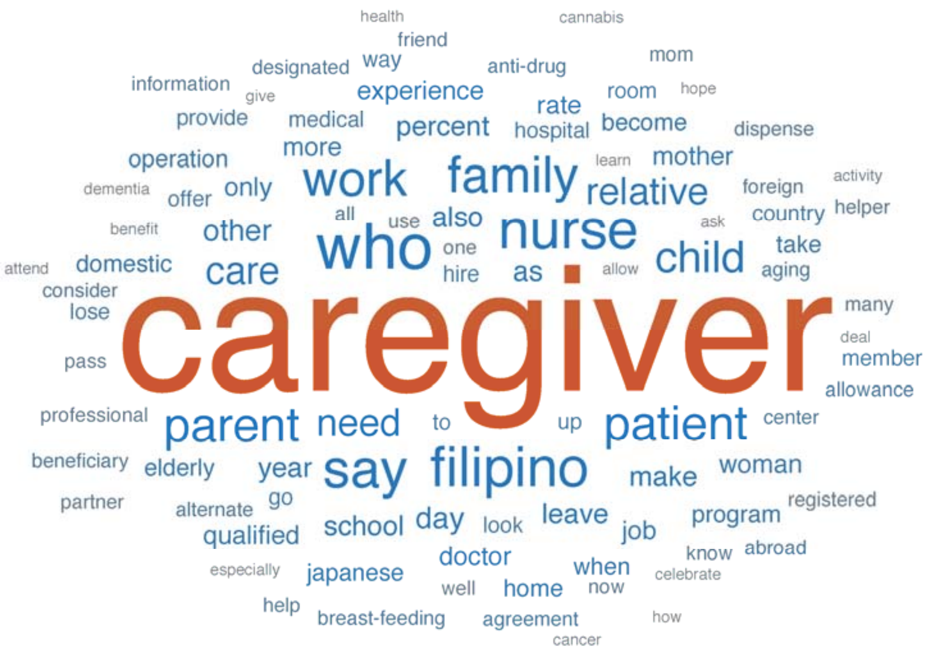

Figure S4. Word cloud which visualizes the words that co-occurred most frequently with ‘caregiver(s)’ in the Philippines.

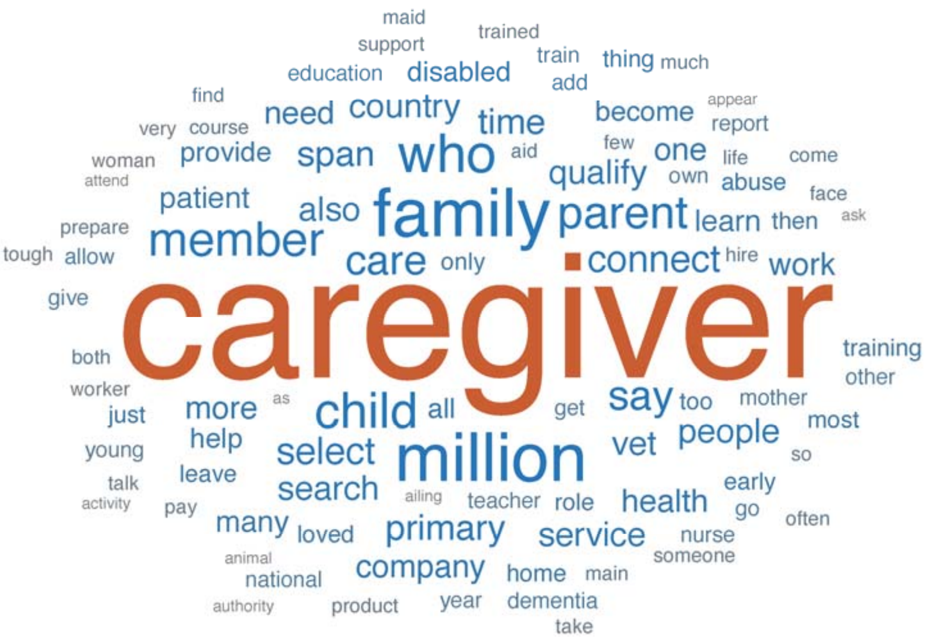

Figure S5. Word cloud which visualizes the words that co-occurred most frequently with ‘caregiver(s)’ in Malaysia.

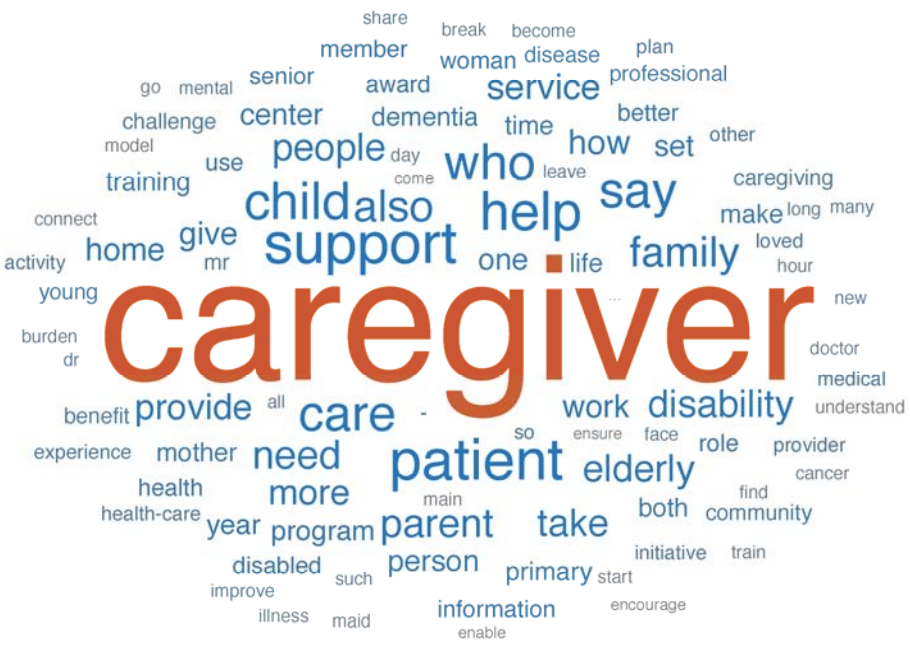

Figure S6. Word cloud which visualizes the words that co-occurred most frequently with 'caregiver(s)' in Singapore.
